# 

**Published:** 1907-11

**Authors:** 


### CONTENTS.

ORIGINAL COMMUNICATIONS—

What You Cannot Do With Purgatives, By Edwin Walker, M. D. Evansville, Ind. ---- 447
Some Anamalies of the Stigmata of Degeneracy, By Marc Ray Hughes, M. D., St. Louis, Mo. ---- 454
A Plea for Greater Accuracy in Our Therapeutics, By J. Robert Buchanan, M. D., Nevada, Mo. ---- 462
"The Frequent Interdependence of Dislocated Kidney, Gall-Bladder Trouble and Appendicitis, By Earl Harlan, M. D., Cincinnati, O. ---- 467
Sub-Mucus Window Resection of the Oartilagious Septum, By R. H. T. Mann, M. D., Texarkana, Ark-Tex. 469
Acquired Syphilis, By W. E. Hughes, Pocahontas, Ark. ---- 472
The Disposition of the Appendiceal Stump, By Benjamin Merrill Rickets. Ph. B., M. D., LL. D., Cincinnati, O. ---- 474

EDITORIAL NOTES AND COMMENTS—

Counter Prescribing ---- 476

NEWS AND NOTES ---- 427

BOOK REVIEWS ---- 434

Progressive Medicine, Vol. III, September, 1907 ---- 480
The Diagnosis and Treatment of Diseases of Women, By Harry Sturgeon Crossen, M. D., St. Louis, Mo, 481

SELECTIONS AND ABSTRACTS—

Inconsistency, Thou art—A State Medical Journal ---- 484
Perineal Section for Obstruction of Prostatic Urethra ---- 482
Rectal Anaesthesia ---- 486
Fehling Test for Sugar ---- 487
The Rational Treatment of Pulmonary Hemorrhage ---- 488
The Pernicious Tendencies of Rampant Organization ---- 484

MEDICAL ITEMS

Syphilo-Dermo-Urologic Maxims ---- 491
Eighth Annual Meeting of the National Association for the Study of Epilepsy and the care and treatment of Epileptics ---- 494
Antiphlogistine Versus Opium ---- 466
The Care of Growing Girls ---- 497
The Pathology and Treatment of Hay Fever ---- 498
Asthma ---- H-M-C And a Happy Delivery ---- 500
Dr. Pettey's Eastern Retreat ---- 504

A New Mystery Story—"Under The Black Cassock." ---- 508

